# Development of the diabetes typology model for discerning Type 2 diabetes mellitus with national survey data

**DOI:** 10.1371/journal.pone.0173103

**Published:** 2017-03-02

**Authors:** Anna Bellatorre, Sharon H. Jackson, Kelvin Choi

**Affiliations:** Division of Intramural Research, National Institute on Minority Health and Health Disparities, Bethesda, Maryland, United States of America; University of Rochester, UNITED STATES

## Abstract

**Objective:**

To classify individuals with diabetes mellitus (DM) into DM subtypes using population-based studies.

**Design:**

Population-based survey

**Setting:**

Individuals participated in 2003–2004, 2005–2006, or 2009–2010 the National Health and Nutrition Examination Survey (NHANES), and 2010 Coronary Artery Risk Development in Young Adults (CARDIA) survey (research materials obtained from the National Heart, Lung, and Blood Institute Biologic Specimen and Data Repository Information Coordinating Center)

**Participants:**

3084, 3040 and 3318 US adults from the 2003–2004, 2005–2006 and 2009–2010 NHANES samples respectively, and 5,115 US adults in the CARDIA cohort

**Primary outcome measures:**

We proposed the Diabetes Typology Model (DTM) through the use of six composite measures based on the Homeostatic Model Assessment (HOMA-IR, HOMA-%β, high HOMA-%S), insulin and glucose levels, and body mass index and conducted latent class analyses to empirically classify individuals into different classes.

**Results:**

Three empirical latent classes consistently emerged across studies (entropy = 0.81–0.998). These three classes were likely Type 1 DM, likely Type 2 DM, and atypical DM. The classification has high sensitivity (75.5%), specificity (83.3%), and positive predictive value (97.4%) when validated against C-peptide level. Correlates of Type 2 DM were significantly associated with model-identified Type 2 DM. Compared to regression analysis on known correlates of Type 2 DM using all diabetes cases as outcomes, using DTM to remove likely Type 1 DM and atypical DM cases results in a 2.5–5.3% r-square improvement in the regression analysis, as well as model fits as indicated by significant improvement in -2 log likelihood (p<0.01). Lastly, model-defined likely Type 2 DM was significantly associated with known correlates of Type 2 DM (e.g., age, waist circumference), which provide additional validation of the DTM-defined classes.

**Conclusions:**

Our Diabetes Typology Model reflects a promising first step toward discerning likely DM types from population-based data. This novel tool will improve how large population-based studies can be used to examine behavioral and environmental factors associated with different types of DM.

## Introduction

Diabetes mellitus (DM) is a public health concern in the US. It has been estimated that 9.3% of the US population (29.1 million) have DM; of those, 27.8% are undiagnosed [1]. DM was the seventh leading cause of death in the US in 2010, claiming 69,071 lives [1]. DM is a complex metabolic disorder that develops due to inadequate insulin production or ineffective insulin utilization by insulin target cells in muscle, fat and the liver. Patients with diabetes are typically classified as having Type 1 (T1DM), Type 2 (T2DM) or gestational diabetes based on the lack of insulin production, insulin resistance or insulin resistance during pregnancy, respectively. Although T2DM is most common, paradoxically some patients manifest symptoms of both T1DM and T2DM. Additionally, other rarer forms of diabetes occur because of specific genetic mutations and pancreatic disease owing to tissue insults from drugs and toxins. Although the incidence of T1DM is highest among children and young adults, it is an autoimmune disease that can manifest at any age [2]. Owing in part to the global obesity epidemic, the incidence of T2DM in children continues to increase, and minority youth are disproportionately affected [3–5].

A current challenge in diabetes research is to use population-based studies to estimate the prevalence of diabetes subtypes despite the imprecise nature of the classification of diabetes in these studies. Respondents are often asked about whether they have ever been diagnosed with diabetes, but are not often asked a follow up question regarding DM type. Further, individuals with undiagnosed DM will not be able to provide information on diabetes subtypes. Additionally, no large national surveys of adults that measured autoantibodies that can be used to identify T1DM cases. In contrast, it is increasingly common for population-based studies to collect physiologic data, including blood glucose and insulin levels that can be used to screen for diabetes and evaluate insulin resistance and sensitivity. Surrogate indicators for insulin resistance and sensitivity, as well as pancreatic β-cell function, can be extrapolated from fasting blood glucose and insulin levels that are commonly included in population-based studies. The homeostatic model assessments (HOMA) are well recognized methods for estimating pancreatic β-cell function and how well insulin is utilized by its target cell populations. Specifically, HOMA-%β is a surrogate for pancreatic β-cell insulin production, HOMA-IR is a measure for insulin resistance, and HOMA-%S is a measure for insulin sensitivity [6, 7]. While we cannot use these surrogate indexes to diagnose DM subtypes, it is possible that we can use these surrogate indexes coupled with anthropometric measures like body mass index (BMI) to correctly classify individuals with DM, both diagnosed and undiagnosed, into their corresponding subtypes. No studies to date have leveraged these measures to perform DM subtype classification.

In this study, we proposed the Diabetes Typography Model (DTM), which aimed to classify individuals with DM into subtypes of DM using a latent class analysis approach. We then examined the sensitivity, specificity, and positive predictive value of this classification method. Lastly, we tested these model-defined classes using known correlates of T2DM to examine the construct validity of these classes in discerning different subtypes of DM. The proposed DTM will enable researchers to estimate prevalence of the various subtypes of DM and to examine the behavioral and environmental factors associated with each subtype of DM in population-based studies.

## Materials and methods

### Data sources

The data analyzed come from 2003–2004, 2005–2006, and 2009–2010 samples of the National Health and Nutrition Examination Survey (NHANES) and from year 25 (2010, Wave 8) of the Coronary Artery Risk Development in Young Adults survey (CARDIA). Respondents with diabetes ranged from 270 (NHANES 2005/6) to 451 (CARDIA 2010) representing 9–13% of each cohort.

The NHANES is a serial cross-sectional health survey of a US representative sample of adults and children undertaken by the U.S. Centers for Disease Control and Prevention [8]. It includes a detailed survey component and full medical examination using a mobile examination center. Data collected include self-reported demographic; social, health and nutrition information; supplementary blood test results and anthropometric measurements. NHANES includes Mexican American, non-Mexican Hispanic, non-Hispanic White, non-Hispanic Black, and participants of other races.

The CARDIA study, began in 1985–1986 with 5,115 adults aged 18–30, is a prospective longitudinal study evaluating the risk of developing heart diseases over time for Black and White US adults [9]. The same cohort of respondents has been followed for eight waves through 2010 (3,450 respondents, ages 43–55) at varying intervals ranging from 1–5 years between waves. Like NHANES, CARDIA collects biological and survey data. The data we used are from Wave 8 collected in 2010. This was a secondary data analysis on de-identified data and therefore was exempted from a review by the institutional review board.

### Measures

#### DTM Model Variables

Due to the importance of the Homeostasis Model in discerning diabetes type [7], we included three measures based in this model- HOMA-IR, HOMA-%β, and HOMA-%S. Due to the importance of insulin levels in these calculations, we omitted all respondents currently taking insulin as the presence of exogenous insulin would affect the validity of the models. HOMA-IR estimates insulin resistance (IR, Eq 1). Higher HOMA-IR values reflect higher IR where the body is producing enough insulin, but the insulin produced is not effectively controlling blood glucose levels; a characteristic of T2DM [10]. A value of 3 indicates moderate insulin resistance and ≥ 5 indicates severe insulin resistance [10]. We use a cut point of 1.7 to classify respondents as having had low HOMA-IR (0–1.7 = 1; else = 0).

$$HOMA-IR=\left(\frac{fasting\;insulin\left(\frac{\mu U}{mL}\right)\times fasting\;glu\cos e\left(\frac{mmol}{L}\right)}{22.5}\right)$$ (1)

HOMA-%β estimates pancreatic β cells’ insulin production function (Eq 2). Insulin production is normal-high in T2DM and low-absent in T1DM. [11] Calculated values 81.7% or lower were classified as low HOMA-%β (0–81.7 = 1; else = 0).

$$HOMA-\%\beta =\left(\frac{fasting\;glu\cos e\left(\frac{mmol}{L}\right)\times 20}{fasting\;glu\cos e\left(\frac{mmol}{L}\right)-3.5}\right)\times 100\%$$ (2)

HOMA-%S estimates insulin sensitivity (Eq 3). High insulin sensitivity indicates the body is utilizing insulin effectively. High insulin sensitivity values are uncommon in T2DM and are more common in T1DM. Respondents with a value of 65% or above were classified as having high HOMA-%S.

$$HOMA-\%S=\left(\frac{1}{\frac{fasting\;insulin\left(\frac{\mu U}{mL}\right)\times fasting\;glu\cos e\left(\frac{mmol}{L}\right)}{22.5}}\right)\times 100\%$$ (3)

Past research has shown a strong relationship between being overweight and obese according to body mass index (BMI, Eq 4) and risk of T2DM [12, 13]. Respondents were classified as low-normal BMI if their BMI was <25.

$$BMI=\left(\frac{weight\left(kg\right)}{height{\left(m\right)}^{2}}\right)$$ (4)

The remaining two measures focus on insulin: (1) low fasting insulin (0–5 μU/mL = 1; else = 0), which is uncommon in T2DM and frequently seen with T1DM and other types of diabetes; [14] and (2) high glucose to insulin ratio (G: I, Eq 5). Type 2-diabetics typically have relatively low G:I ratios because they have high insulin production relative to glucose levels. The opposite pattern is typically seen with T1DM. Respondents with values >20 were classified as having high G:I ratio.

$$Glu\cos e\;to\;insulin\;ratio=\left(\frac{fasting\;glu\cos e\left(\frac{mg}{dL}\right)}{fasting\;insulin\left(\frac{\mu U}{mL}\right)}\right)$$ (5)

#### Demographic and T2DM correlates

Both NHANES and CARDIA sample different racial/ethnic groups. For CARDIA data, we include both Black and White respondents. For NHANES data, we re-categorized racial/ethnic categories to Hispanic (of any origin), non-Hispanic Black, non-Hispanic White, and other. Other demographic variables included sex (male = 1; female = 0), age at interview (continuous), marital status (married = 1; else = 0), and level of education (high school or less, some college, or college or above). We included the following six dichotomous measures of known T2DM correlates in our analyses: high gender-specific waist circumference (female—35+ inches, male 40+ inches), severe insulin resistance (HOMA-IR = 5+), high triglycerides (200+), high total cholesterol (200+), high diastolic blood pressure (90+ mmHg), and high systolic blood pressure (140+ mmHg).

### Statistical analysis

Our focal analyses involve latent class analyses (LCA) across four samples of diabetic respondents identified either through self-report (diagnosed) or by hemoglobin A1c (HA1c) level (undiagnosed) in each sample of individuals. LCA is a type of mixture model, which is developed to explore the heterogeneity within a population that is not directly observed. LCA aims to categorize individuals into mutually exclusive and exhaustive subpopulations (i.e., classes), based on their responses to a set of directly observed variables, so that each class will present a unique pattern of responses. “Best” model was chosen based on entropy, model fit, interpretability, and class sizes. Goodness of fit was tested using Vuong-Lo-Mendell-Rubin Likelihood Ratio Test to examine if a k-class model was a better fit than a k-1 class model, in which a significant (p<0.05) test results means the model with higher number of classes is a better fit than the one with lower number of classes. Six indicators (HOMA-IR, HOMA-%β, HOMA-%S, BMI, glucose to insulin ratio, and fasting insulin) were included in the LCA models. The analysis was conducted across four different samples to determine if the best-fit model is replicated across samples. Mplus version 7.4 was used to conduct the latent class analysis. Class membership based on the “best” model was then assigned to each individual in each sample.

To validate the empirically derived classes, we cross-tabulated class membership against whether respondents had C-peptide values consistent with LCA defined classes. Low C-peptide is a T1DM correlate that quantifies endogenous insulin secretion [15, 16], which would be low in T1DM and normal/high in T2DM cases. C-peptide measurements were only available in the 2003–2004 NHANES data. We calculated positive predictive value, sensitivity, and specificity of DTM in this sample. We also examined whether excluding DTM-defined cases that were inconsistent with the physiological profile of T2DM would increase the model fit and variance explained when assessing the association between demographics and known T2DM correlates across studies. For these analyses, we ran logistic regression models predicting all diabetes against logistic regression models predicting DTM-defined likely T2DM. Model fit statistics (variance explained, pseudo r-square, negative 2-log likelihood) were examined.

## Results

Table 1 displays means or proportions of relevant values for measures included in the proposed DTM, demographics, and diabetes correlates for each sample for both the full sample and all diabetics in the sample. As shown, the prevalence of low fasting insulin, low/normal BMI, and high G:I ratios progressively decrease between 2003–2004 and 2009–2010. However, among all diabetics, the HOMA indicators did not show a clear trend between 2004 and 2010.

**Table 1:** Means or percentages of relevant variables in NHANES 2003–2004, 2005–2006, and 2009–2010 & CARDIA 2010.

	NHANES 2003–2004		NHANES 2005–2006		NHANES 2009–2010		CARDIA 2010	
	Full Sample	All Diabetics	Full Sample	All Diabetics	Full Sample	All Diabetics	Full Sample	All Diabetics
***Measures Included in the LCA Model***	*** ***	*** ***	*** ***	*** ***	*** ***	*** ***	*** ***	*** ***
*Insulin Resistance*	* *	* *	* *	* *	* *	* *	* *	* *
Low HOMA-IR*	43%	13%	36%	17%	27%	10%	41%	11%
*Beta Cell Function*	* *	* *	* *	* *	* *	* *	* *	* *
Low HOMA-%β*	41%	62%	39%	66%	20%	51%	37%	46%
*Insulin Sensitivity*	* *	* *	* *	* *	* *	* *	* *	* *
High HOMA-S%*	38%	12%	31%	15%	22%	9%	36%	10%
*Insulin and Glucose*								
Fasting Insulin below 5 μU/mL*	25%	14%	19%	14%	10%	8%	23%	7%
Glucose to Insulin Ratio 20+*	22%	25%	17%	28%	8%	19%	19%	14%
*Body Mass Index*								
Low or Normal BMI*	42%	16%	41%	15%	35%	12%	25%	8%
***Demographics***	** **	** **	** **	** **	** **	** **	** **	** **
Hispanic	26%	33%	27%	24%	33%	36%		
NH White	44%	39%	43%	40%	45%	39%	54%	34%
NH Black	25%	23%	26%	33%	17%	19%	46%	66%
Other Race	4%	5%	4%	3%	6%	6%		
Female	50%	50%	51%	54%	52%	48%	56%	59%
Male	50%	50%	49%	46%	48%	52%	44%	41%
***Diabetes Correlates and Indicators***								
Glycohemoglobin (%)	5.49	7.42	5.44	7.52	5.66	7.37	5.71	7.34
Hemoglobin A1C 10+	1%	9%	1%	13%	1%	9%	1%	11%
Gender-Adjusted Waist Circumference	91.00	101.99	92.17	105.49	93.52	103.93	94.25	105.42
High Triglycerides	11%	25%	14%	31%	11%	24%	9%	21%
Total Cholesterol 200+	36%	46%	35%	39%	36%	33%	29%	28%
High Diastolic Blood Pressure	3%	4%	3%	6%	4%	7%	8%	11%
High Systolic Blood Pressure	13%	27%	12%	27%	13%	30%	9%	16%

**Notes**:

* Indicates specific dichotomous measure used in latent class analysis models

### Latent class analyses

Table 2 displays results of the latent class analyses. The percentages under each class represent the proportion of respondents in that particular class having a specific attribute. For example, in 2003–2004 NHANES, 100%, 1.5%, and 4.1% of respondents in Class 1, 2, and 3, respectively, had HOMA-IR lower than 1.7. We also presented the size of each class and its proportion to all diabetes respondents in a given sample. Three of the four models had entropy values above 0.995 with the fourth (NHANES 2009–2010 sample) at 0.817, indicating high classification certainty. Both Vuong-Lo-Mendell-Rubin tests and Lo-Mendell-Rubin adjust likelihood ratio tests showed a three-class model fitted the data significantly better than a two-class model in all samples (*P*<0.0001), while a four-class model did not significantly fit the data better than a three-class model (*P*>0.05).

**Table 2:** Latent class analysis of the Diabetes Typology Model.

Diabetes Correlate Cut Point	2003–2004 NHANES			2005–2006 NHANES			2009–2010 NHANES			2010 Cardia		
	Class 1	Class 2	Class3	Class 1	Class 2	Class3	Class 1	Class 2	Class3	Class 1	Class 2	Class3
HOMA-IR 1.7 or Lower	100.00%	1.50%	4.10%	100.00%	1.50%	0.00%	100.00%	0.00%	2.50%	100.00%	1.90%	0.00%
HOMA-%β 81.7 or Lower	96.90%	49.00%	100.00%	88.70%	51.50%	100.00%	78.50%	24.20%	100.00%	72.40%	36.70%	100.00%
HOMA-%S 65+	99.10%	0.00%	0.00%	90.20%	0.00%	0.00%	94.20%	0.00%	0.00%	97.10%	0.00%	0.00%
BMI 25 or Lower	46.40%	10.40%	19.20%	36.10%	6.10%	29.50%	40.20%	2.80%	21.30%	29.80%	5.20%	15.90%
Glucose to Insulin Ratio above 20	81.40%	0.00%	100.00%	72.10%	0.00%	100.00%	70.00%	0.00%	43.00%	45.20%	0.00%	100.00%
Fasting Insulin Below 5	90.70%	0.00%	20.90%	74.40%	0.00%	13.60%	70.00%	0.00%	3.60%	63.20%	0.00%	11.40%
Number in Estimated Class	32	202	42	44	182	44	37	289	68	44	363	44
Percentage of Diabetics in Sample	11.59%	73.19%	15.22%	16.30%	67.41%	16.30%	9.39%	73.35%	17.26%	9.76%	80.49%	9.76%
**Model Fit 3 Classes**												
Entropy		0.998			0.996			0.817			0.998	
Diabetic total that year		276			270			394			451	
Vuong-Lo-Mendell-Rubin P-Value (3-class versus 2-class model)		0.0000			0.0000			0.0000			0.0000	
Lo-Mendell-Rubin Adj. LRT P-Value (3-class versus 2-class model)		0.0000			0.0000			0.0000			0.0000	

**Fig 1:**
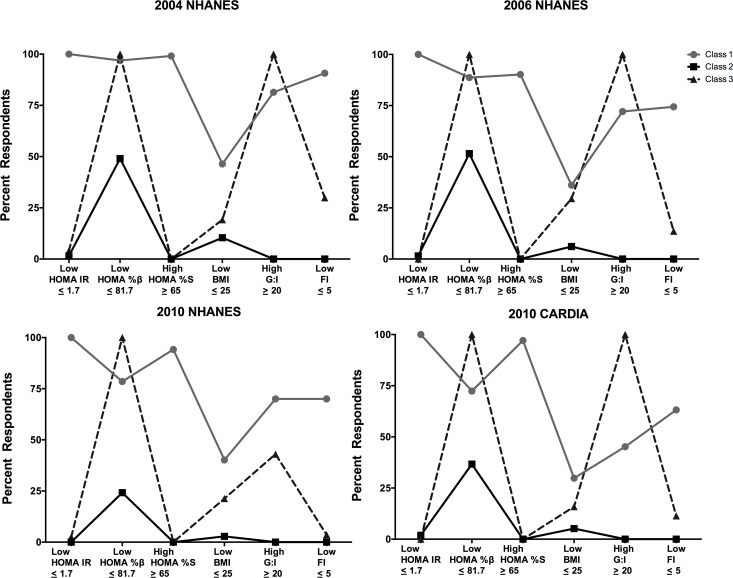
Latent class analysis of all diabetic respondents in the study samples indicated, by the diabetes correlates on the X-axis: National Health and Nutrition Examination Survey (NHANES) and the Coronary Artery Risk Development in Young Adults survey (CARDIA).

The profiles of these three classes were also consistent across samples. Fig 1 presents the percent of respondents in each class having each of the six indicators. Class 1 is characterized by uniformly low HOMA-IR, high prevalence of low HOMA-%β, high prevalence of high HOMA-%S, moderate-high prevalence of high G:I ratios, high prevalence of low fasting insulin. The measurement profile of this class was consistent with T1DM. We named this class “likely T1DM”. Class 2 was characterized by uniformly low prevalence of high HOMA-%S, low prevalence of low fasting insulin, high G:I ratios; low prevalence of low HOMA-IR, and low prevalence of low or normal BMI. The measurement profile of this class was consistent with T2DM. We named this class “likely T2DM”.

A consistent third class with a unique measurement profile also exists among individuals with DM. Class 3 had uniformly high prevalence of low HOMA-%β and low prevalence of high HOMA-%S, moderate-high prevalence of high G:I ratios, very low prevalence of low HOMA-IR, low prevalence of low or normal BMI, and low prevalence of low fasting insulin. Since the measurement profile of this class was inconsistent with either T1DM or T2DM, we named this class “atypical DM”.

### Validation analyses

The top part of Table 3 presents the validation analysis of DTM-defined T2DM against C-peptide. When cross-tabulating C-peptide (low vs. normal/high) and class membership (class 2 vs. other) among diabetics not taking insulin in the 2003–2004 NHANES data, the DTM has 75.5% sensitivity and 83.3% specificity for T2DM, indicating it identified 75.5% of all cases with normal/high C-peptide as likely to be T2DM and 83.3% of all cases with low C-peptide as unlikely to be T2DM (Table 3). More importantly however, is that the positive predictive value of the DTM is 97.42%, which shows that 97.42% of the model-identified T2DM cases had normal or high C-peptide. This further demonstrates that the DTM can classify T2DM with a high level of certainty.

**Table 3:** Sensitivity analyses using C-peptide matching and increased variance explained.

	2003–2004 NHANES			2005–2006 NHANES			2009–2010 NHANES			2010 CARDIA		
	Class 1	Class 2	Class 3	Class 1	Class 2	Class 3	Class 1	Class 2	Class 3	Class 1	Class 2	Class 3
**Sensitivity Analysis 1. C-Peptide Matching**^a^												
Positive Predictive Value of C-Peptide and not on Insulin		97.42%										
Sensitivity with C-Peptide and not on Insulin		75.50%										
Specificity with C-Peptide and not on Insulin		83.33%										
**Sensitivity Analysis 2. Variance Explained Using Classes**^b^												
Predicting All Diabetes Cases (1 = Diabetes, 0 = No Diabetes)		0.3197			0.2990			0.2466			0.1898	
LCA Defined Class (1 = Class, 0 = Else)	0.2161	0.3549	0.1882	0.1390	0.3519	0.1476	0.1288	0.2729	0.1554	0.0364	0.2380	0.1213
Best Measure		**Class 2**	** **	** **	**Class 2**	** **	** **	**Class 2**	** **	** **	**Class 2**	
**Model Fit**												
Increase in R-Squared Using Best Class		0.0352			0.0529			0.0263			0.0482	
Negative 2 Log Likelihood of All Diabetes Model		-644.64			-655.94			-930.38			-1083.92	
Negative 2 Log Likelihood of Likely Type 2 Diabetes Model		-483.06			-447.93			-685.84			-884.31	
Model Degrees of Freedom		14			14			14			12	
Improvement in -2 Log Likelihood		161.58			208.01			244.54			199.61	
P-Value of Improvement		0.0000			0.0000			0.0000			0.0000	

**Notes**:

a. C-peptide values only available in 2003–2004

b. Model includes: Race, gender, age, marital status, education level, gender adjusted waist circumference, high total cholesterol, high triglycerides, high diastolic blood pressure, high systolic blood pressure, and an indicator for severe insulin resistance.

The bottom part of Table 3 presents variance explained and various model fit statistics from regression models with all DM cases versus regression models with only DTM-defined T2DM cases. When comparing the variance explained by known correlates of T2DM, we found that excluding DTM-defined unlikely T2DM cases (i.e., classes 1 and 3) increased the variance explained by known correlates in multiple logistic regression models. Specifically, variance explained was 19%-32% using all cases of diabetes in the regression models, and increased to 24%-35% (a 2.6%-5.3% increase in variance explained; Table 3). Similarly, we observed significant improvement in model fit (i.e., -2-log likelihood after excluding unlikely T2DM cases) from the models.

Table 4 shows the associations between known T2DM correlates and DTM-define T2DM (Class 2), after removing DTM-defined non-T2DM (classes 1 and 3). Significant positive associations were observed between Class 2 (vs. non-diabetics) and known T2DM risk factors: non-White race/ethnicity, older age, high gender-adjusted waist circumference, severe insulin resistance, and high triglycerides.

**Table 4:** Detailed logistic regression predicting model-defined Type 2 Diabetes Mellitus (Class 2) using LCA defined classes of NHANES & CARDIA Data.

	NHANES 2003–2004		NHANES 2005–2006		NHANES 2009–2010		CARDIA 2010	
	AOR (95% CI)		AOR (95% CI)		AOR (95% CI)		AOR (95% CI)	
*Demographics*	* *	* *	* *	* *	* *	* *	* *	* *
Hispanic	1.80 (1.16,2.79)	**	2.06 (1.25,3.41)	**	2.67 (1.89,3.78)	***		
NH Black	1.91 (1.20,3.04)	**	3.70 (2.38,5.76)	***	2.32 (1.54,3.49)	***	2.09 (1.58,2.78)	***
Other Race	2.93 (1.29,6.67)	*	0.79 (0.22,2.78)		2.22 (1.12,4.40)	*		
Male	1.03 (0.72,1.46)		0.47 (0.32,0.69)	***	0.71 (0.53,0.95)	*	0.55 (0.42,0.71)	***
Age at Interview	1.06 (1.05,1.07)	***	1.07 (1.06,1.08)	***	1.06 (1.05,1.07)	***	1.06 (1.03,1.10)	***
Married	1.25 (0.87,1.81)		1.56 (1.08,2.26)	*	1.44 (1.07,1.93)	*	1.20 (0.93,1.56)	
High School or Less	1.09 (0.64,1.84)		1.13 (0.65,1.98)		1.42 (0.90,2.23)		1.20 (0.87,1.66)	
Some College	1.06 (0.58,1.94)		1.02 (0.55,1.90)		1.67 (1.02,2.73)	*	1.20 (0.89,1.61)	
*Known Type 2 Diabetes Correlates*								
High Gender-Adjusted Waist Circumference	1.01 (1.00,1.02)	**	1.02 (1.01,1.03)	***	1.02 (1.01,1.03)	***	1.05 (1.04,1.06)	***
Severe Insulin Resistance	14.70 (10.21,21.18)	***	6.00 (4.05,8.89)	***	4.99 (3.68,6.76)	***	4.93 (3.72,6.53)	***
High Triglycerides	1.89 (1.25,2.85)	**	2.64 (1.75,3.99)	***	2.13 (1.49,3.04)	***	2.14 (1.51,3.03)	***
Total Cholesterol 200+	0.84 (0.59,1.20)		0.55 (0.37,0.80)	**	0.57 (0.42,0.77)	***	0.93 (0.71,1.23)	
High Diastolic Blood Pressure	0.80 (0.33,1.94)		1.35 (0.64,2.82)		1.25 (0.69,2.25)		0.69 (0.43,1.11)	
High Systolic Blood Pressure	0.92 (0.60,1.40)		1.00 (0.66,1.54)		1.30 (0.92,1.83)		1.42 (0.92,2.19)	
Model Fit Indicies								
LR Chi-Squared Value	531.59		486.43		514.93		554.95	
LR Chi-Squared Value Degrees of Freedom	14		14		14		12	
Prob >Chi-Squared	0.0000		0.0000		0.0000		0.0000	
Psuedo R Squared	0.3549		0.3519		0.2729		0.2338	
N	3084		3040		3318		3450	

Notes:

*P<0.05

**P<0.01

***P<0.001

AOR = Adjusted odds ratios, CI = Confidence interval, NH-non-Hispanic. Reference group: Race/ethnicity = non-Hispanic White; gender = female; marital status = not married; education = college graduate; waist circumference = normal or low gender-adjusted waist circumference; insulin resistance = HOMA-IR<5; triglycerides = triglycerides<200; total cholesterol = total cholesterol<200; diastolic blood pressure = diastolic blood pressure <90mmHg; systolic blood pressure = systolic blood pressure <140mmHg.

## Discussion

Existing research on prevalence, incidence, and predictors of DM type across the life course is often limited to small clinical samples of diagnosed T1DM or T2DM cases, [17–19] or samples restricted to one stage of the life course [20]. Population-based research can inform trends on DM prevalence and incidence among diagnosed and undiagnosed diabetics. However, the inability to classify diabetes subtype in population-based studies hinders researchers from tracking DM prevalence, and from using population-based studies to examine risk factors for each subtype of DM.

Even though some studies, such as SEARCH for Diabetes in Youth, [20, 21] are able to classify diabetes subtype from a blood sample and testing for autoantibodies, this ability comes with high costs and is not standard practice for adult population-based surveys without an expressed diabetes focus. It is expensive and logistically challenging to have blood drawn via venipuncture for all respondents in a large national sample. Additionally, sending phlebotomists to individuals’ homes is costly and practically cumbersome. Alternatively, asking individuals to visit a clinic for a blood draw is likely to result in low response rate, particularly among those with limited access to medical care including residents in rural areas and individuals of low socioeconomic status. Due to the challenges of collecting blood samples by venipuncture, some nationally representative studies have shifted toward blood collection via blood spots especially for glucose and HbA1c testing [22].

Given the utility of a method to sort population-based samples of DM by subtype, we sought to create a model that could use multiple sources of biological and anthropometric data from population-based data to meet this goal. Moreover, we sought measures that would yield valid indicators from blood samples collected by either venipuncture or blood spot methods. As demonstrated here the DTM, based on an expanded set of variables and measures of the Homeostatic Model, is capable of meeting this goal. Model fit statistics showed that the DTM possesses a high certainty in assigning individuals with diabetes into three classes (likely T1DM, likely T2DM, atypical DM). Our validation analyses also showed that DTM has a high positive predictive value, sensitivity, and specificity in identifying DM cases that are consistent with the physiological and anthropometrical profile of T2DM among individuals with diabetes. Herein we also showed that by excluding model-defined classes 1 and 3, known T2DM correlates (e.g., non-White race/ethnicity, older age, high gender-adjusted waist circumference, severe insulin resistance, and high triglycerides) explained a greater amount of variance for class 2 (T2DM) than unsorted comingled DM cases. Given that the physiological measures used in LCA can be obtained through blood spots, DTM greatly increases our ability to separate DM type in large population-based studies, thus enhancing our ability to examine the risk factors for each type of DM outside of clinical studies.

Interestingly, DTM consistently detects a third class of individuals with DM that did not fit the T1DM-T2DM dichotomy. The size of this class (10%-17% across four samples of individuals) is non-trivial among all diabetics and additional investigations are needed to determine the significance of this DTM identified population. While the available data is insufficient to determine whether this group reflects a different subtype of DM, we suspect that it could be an atypical presentation of a variant or subtype of either T1DM or T2DM, e.g., Maturity Onset Diabetes of the young (MODY) or secondary diabetes. However, we cannot conclusively determine this with our data. Nonetheless, the finding that a clear third class emerged from the use of the DTM is promising given that the model clearly functions with its intended purpose of distinguishing different subtypes of DM, which would make research on this atypical DM variant possible in future studies.

As prevalence of all subtypes of DM increase and present across the age spectrum, it will become increasingly important for population-based research to identify social and environmental precursors to the development of DM. The only information required to replicate our latent class analyses are respondent DM status (either self-report or confirmed using HbA1c levels), body mass index (or both height and weight), and fasting glucose and fasting insulin levels, which can be collected by either venipuncture or blood spot. Thus, the simplicity of this model and general availability of these measures in population-based studies employing select biological sample collections creates a unique and important opportunity to begin more comprehensive research on the incidence, prevalence, and socio-environmental contexts of DM by subtype.

Despite DTM’s ability to classify DM subtypes in population-based studies, our analysis has several limitations. First, DTM cannot be used in pre-diabetic or normoglycemic individuals. However, once the presence of diabetes has been established, this model can facilitate sorting of cases by likely diabetes subtype. Second, while we were able to replicate the model in four different national adult samples, the utility of DTM in specific subpopulations is unknown. Future research needs to focus on refining this model and testing its accuracy across the age spectrum and race/ethnicity. Third, we were only able to validate DTM-defined likely T2DM classification with C-peptide in one of the four samples since the measure was not available in the other three samples. Measures to validate DTM-defined likely T1DM and atypical DM were not available in NHANES and CARDIA. A previous analysis estimated the prevalence of T1DM based on age of DM diagnosis, age of insulin initiation, and current use of insulin. [23] However, with the increasing prevalence of late onset T1DM and early onset T2DM resulting in early insulin use, the previous approach may misclassify DM subtypes and should not be used to validate DTM-defined likely T1DM cases. DTM will benefit from additional validation using data from large samples of clinically confirmed T1DM and T2DM cases. This will allow for a more confident determination of the model’s validity and its ability to predict T1DM and T2DM. Lastly, we did not have information about use of oral hypoglycemic agents in our samples. It is possible that some individuals may be using these agents at the time of study. However, we did not find reports in the literature that indicate oral hypoglycemic agents having the ability to change insulin levels and to alter HOMA indexes used in DTM. In conclusion, DTM is a novel tool for classifying DM subtypes in large population-based datasets, and it has great potential to improve how these vast datasets are used to examine behavioral and environmental factors associated with different types of DM. Potential discoveries using this tool can inform preventive clinical practice in the near future.
